# Supplementary material for: Identification of photoperiod-induced specific miRNAs in the adrenal glands of Sunite sheep (Ovis aries)
Source: Front Vet Sci. 2022 Jul 22;9:888207. doi: 10.3389/fvets.2022.888207 (PMC9354845; doi:10.3389/fvets.2022.888207)
Supplement: Supplementary file 1 [file Data_Sheet_1.ZIP › Supplementary file/miRDeep2 file.zip!/miRDeep2 file/Know_Structure/SP42A3/oar-mir-154a.pdf]

miRBase precursor : oar-mir-154a
Total read count : 508
oar-miR-154a-5p read count : 2
oar-miR-154a-3p read count : 505
remaining reads : 1

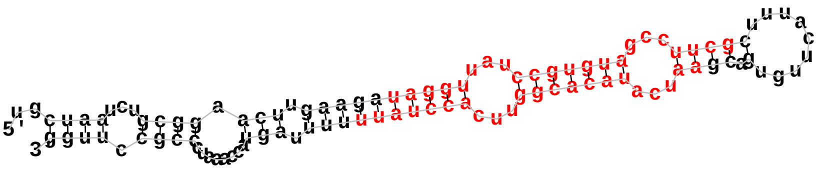

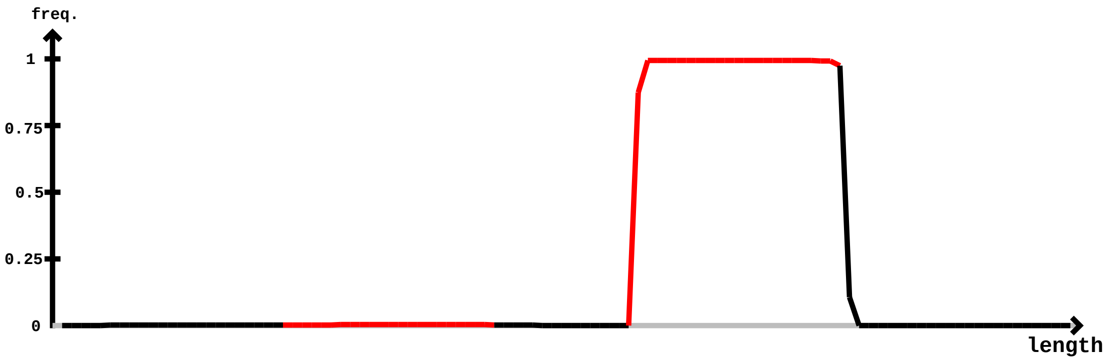

oar-miR-154a-5p

oar-miR-154a-3p

```
5'- ugcuaaucugcggaacuugaagauagguuauccguguagccuucgcuuuacuugugacgaaucauacacgguucaccuauuuuuuaguaccaaaucccgccuugg   -3'    exp
    .((......))((.(((.((((((((((...(((((((...(((((.........).))))...)))))))...))))))))))).))).)).............. reads    mm    sample
    ....aaucugcggaacuugaaga.................................................................................. 1        0      seq
    .......................uagguuauccguguagccuuc............................................................. 1        0      seq
    ............................uauccguguagccuucgcuuu........................................................ 1        0      seq
    ...........................................................aaucauacacgguucaccu........................... 1        0      seq
    ...........................................................aaucauacacgguucaccuau.......................... 9        0      seq
    ...........................................................aaucauacacgguucaccuauu........................ 430      0      seq
    ...........................................................aaucauacacgguucaccuauuuu...................... 4        0      seq
    ............................................................aucauacacgguucaccuauu........................ 11       0      seq
    ............................................................aucauacacgguucaccuauuuu...................... 50       0      seq
```
